# Same‐Day Discharge Metabolic‐Bariatric Surgery in Australia: Experience in a Regional Public Hospital

**DOI:** 10.1111/ans.70289

**Published:** 2025-08-15

**Authors:** Lee S. Kyang, Igor Lemech, Richard Harrison, Denbigh Simond, Nicholas Williams

**Affiliations:** ^1^ Upper Gastrointestinal Surgery Unit, Department of Surgery Wagga Wagga Base Hospital Wagga Wagga New South Wales Australia; ^2^ Faculty of Medicine, University of New South Wales Sydney New South Wales Australia; ^3^ Department of Anaesthesia Wagga Wagga Base Hospital Wagga Wagga New South Wales Australia; ^4^ Rural Clinical School, School of Medicine, The University of Notre Dame Australia Wagga Wagga New South Wales Australia

## Abstract

**Background:**

Access to publicly funded bariatric surgery (MBS) remains suboptimal across Australia and New Zealand. The COVID‐19 pandemic placed additional stress on the public healthcare system, and as a result, overnight beds in public hospitals have become a premium commodity. Same‐day discharge (SDD) MBS has been shown to enhance efficiency and reduce costs in Europe and the United States. This study aims to evaluate the safety and clinical outcomes of implementing SDD MBS in a public regional hospital in Australia.

**Methods:**

A retrospective analysis was conducted using a prospectively maintained database of patients who underwent SDD MBS at Wagga Wagga Base Hospital between December 2018 and September 2024. Patient selection followed strict inclusion criteria and a multidisciplinary approach. Standardised perioperative protocols were applied, with virtual follow‐up at 24–48 h and outpatient intravenous hydration provided if needed. Outcomes included successful SDD rates, 30‐day readmissions, complications, and mortality.

**Results:**

Thirty‐eight patients underwent MBS with intended SDD. Thirty‐five (92.1%) were successfully discharged on the same day. Three (7.9%) patients required overnight hospitalisation. The 30‐day readmission rate was 2.9% (*n* = 1/35), with no postoperative complications or mortality recorded. Outpatient intravenous hydration was required in 11.4% (*n* = 4/35) of cases. Laparoscopic sleeve gastrectomy was the most common procedure (68.4%).

**Conclusion:**

This study demonstrates that SDD MBS is a safe and effective pathway in selected patients, with low readmission and complication rates. Implementing SDD in the public sector has the potential to improve access to bariatric services, reduce healthcare costs, and optimise resource utilisation.

## Graphical abstract

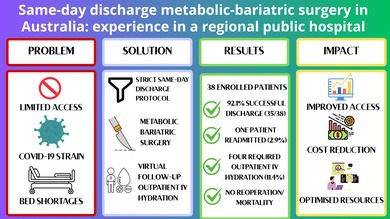

## 1 Introduction

Obesity and overweight are now the main cause of preventable death and disease in Australia, causing 8.3% of the burden of disease in Australia [1]. Metabolic‐bariatric surgery (MBS) is a proven cost‐effective intervention for achieving long‐term weight loss and mitigating chronic diseases [2, 3, 4]. The efficacy and positive outcomes translate at a community clinical level in a recent analysis of The Australia and New Zealand Bariatric Surgery Registry [5]. As obesity prevalence rises, the demand for MBS is expected to increase [6]. Despite this, access to publicly funded MBS remains limited in Australia and Aotearoa New Zealand [7]. In Australia, over 90% of MBS is performed in the private sector. According to the National Bariatric Registry, only 10 out of 22 public hospitals had a significant bariatric caseload, defined as more than 75 cases per year, in 2019 [8]. The COVID‐19 pandemic further strained the public healthcare systems, exacerbating bed shortages in public hospitals [9].

Evidence from Europe and the United States supports the safety and feasibility of same‐day discharge (SDD) after MBS [10, 11, 12, 13]. A recent retrospective analysis of 14 624 patients undergoing SDD sleeve gastrectomy at a high‐volume American centre found no significant difference in readmission rates compared to overnight stays (2.9% vs. 3%, *p* = 0.5) [11]. Similarly, a Seattle study reported readmission and reoperation rates of 2.53% and 1.42%, respectively, among 2534 SDD cases, with a mortality rate of 0.12% [14]. These findings highlight the potential for SDD to fast‐track MBS in selected patients.

Introducing SDD in Australia could yield two major benefits. First, SDD could reduce hospital costs, nosocomial infections, and improve patient satisfaction [15]. A French study demonstrated a 6% cost reduction per patient when sleeve gastrectomy was performed as a day‐case procedure [16]. Second, SDD could enhance access to public sector MBS by optimising financial resources. Wagga Wagga Base Hospital, a rural public hospital in New South Wales, performs approximately 50 MBS annually and is the first in the state to introduce SDD MBS. Initiated in 2022 during the COVID‐19 pandemic, the program employs a multidisciplinary approach and stringent follow‐up protocols. The aim of this study was to report the safety profile and clinical outcomes of SDD MBS in this institution.

## 2 Methods

From a prospectively maintained database between December 2018 and September 2024, this retrospective study included all patients who underwent MBS with intended SDD at a public regional hospital in New South Wales. Patients were excluded if they relocated before follow‐up, had previous MBS, or withdrew consent postoperatively.

### 2.1 Preoperative Selection

Patients referred to the metabolic obesity service (MOS) were triaged using the Edmonton obesity staging system (EOSS) and prioritised for surgery based on clinical need. To qualify for publicly funded MBS, patients had to meet stringent eligibility criteria (Table 1) and complete preoperative workshops and consultations. All candidates underwent preoperative screening conducted by a dedicated bariatric anaesthetist before receiving approval for surgery and SDD.

**TABLE 1:** Inclusion and exclusion criteria for consideration of public‐funded metabolic‐bariatric surgery.

Inclusion criteria
Medicare eligible Patient is willing to consider surgery as a management tool for obesity Recently tried and optimised lifestyle or medical treatments for weight loss that resulted in insufficient weight loss (within the last 2 years) Resides in a rural or remote area of NSW 18–55 years of age Body mass index (BMI) between 40 and 55 kg/m^2^ Patient is willing to participate in a long‐term healthy lifestyle program Non‐smoker or ex‐smoker who has quit for longer than 6 months prior to referral

Exclusion criteria
Previous bariatric surgery Patient is on another public bariatric surgery waitlist Pregnant/planning pregnancy in 2 years post‐surgery History of poor compliance with lifestyle, medical, or mental health interventions Mental health conditions that are not fully treated or stabilised Current alcohol/opioid/illicit drug dependence Cognitive or behavioural disorders without adequate social/carer support

**FIGURE 1:**
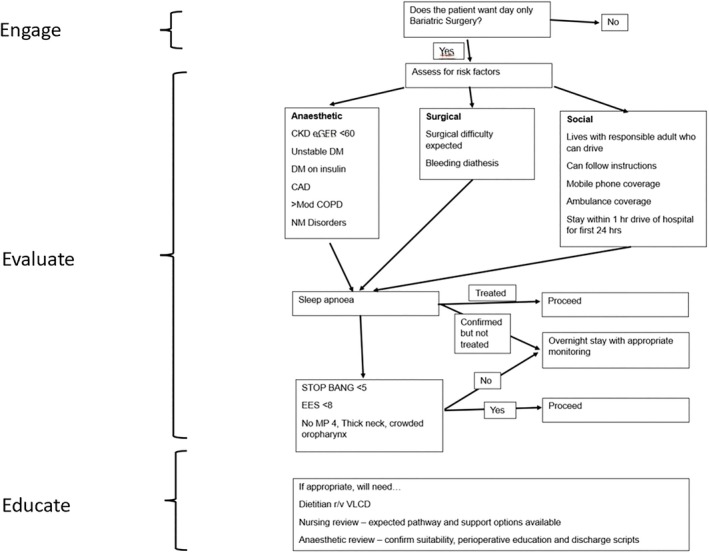
Flowchart illustrating the patient selection process for same‐day discharge.

Suitability and eligibility for day‐only surgery was assessed based on patients' comorbidities and specific selection criteria (Figure 1). Ideal candidates had minimal comorbid conditions, such as well‐controlled diabetes without insulin, minimal coronary artery or renal disease, and no severe respiratory or neuromuscular disorders. Obstructive sleep apnea (OSA) risk was evaluated on a case‐by‐case basis using tools such as the STOP‐BANG questionnaire and the Epworth sleepiness scale. Formal sleep studies were not performed. Additional eligibility requirements included residing within 100 km of the hospital for the first postoperative night, having a responsible adult caregiver with a mobile phone and ambulance coverage, and the ability to follow postoperative instructions.

Selected patients were educated on key aspects of care, including hydration, perioperative pain management, and nausea control. Patients were also informed of specific risks, including the potential for postoperative bleeding and the exacerbation of OSA. Discharge scripts and information were given at a separate day surgery consult to ensure all patient expectations and concerns were met.

### 2.2 Perioperative Protocol

Patients were admitted early on surgery day, and those eligible for SDD were prioritised for morning procedures to allow sufficient postoperative observation. A standardised anaesthetic protocol with an opioid‐free anaesthetic combined with multimodal analgesia was employed. Postoperative care included analgesia, antiemetics, and a minimum one‐month course of proton pump inhibitors (PPIs).

### 2.3 Surgical Techniques

All patients underwent their procedure with three different bariatric surgeons at our institution according to a defined surgical protocol to standardise the operative techniques.

Laparoscopic sleeve gastrectomy (LSG) was performed by dividing the gastrocolic ligament along the greater curvature from the pylorus to the base of the left crus. A tubular stomach was created with sequential firings of a 60 mm linear stapling device starting 2 cm away from the pylorus to 1 cm lateral to the angle of His. A 40 French Bougie was used for calibration. The proximal firings were typically buttressed, and the remainder was oversewn, reattaching gastrocolic omentum.

Laparoscopic Roux‐en‐Y gastric bypass (RYGB) was performed by creating a short gastric pouch 5–6 cm below the oesophago‐gastric junction using a linear stapling device calibrated to a 36Fr Bougie. The gastrojejunostomy and jejuno‐jejunostomy were formed with the same stapling device to achieve a 150 cm alimentary limb and a 50 cm biliopancreatic limb. The defects at gastrojejunostomy, jejuno‐jejunostomy, and both mesenteric windows were closed using barbed sutures.

Laparoscopic one‐anastomosis gastric bypass (OAGB) was performed in the same fashion as RYGB but with a longer pouch commencing at the incisura. The biliopancreatic limb was typically 200 cm long; the afferent loop hitched to the vertical component of the pouch with a running barbed suture, and the large mesenteric defect was not closed.

### 2.4 Same‐Day Discharge

Post‐surgery, patients were initially monitored in recovery before being moved to the day surgery recovery. They were assessed by a MOS nurse practitioner and bariatric surgeon before discharge. Discharge criteria included controlled pain and nausea, adequate hydration, and stable vitals (Table 2). Patients not meeting criteria were admitted overnight and were classified as unsuitable for SDD. Discharged patients received detailed post‐discharge instructions and a 24–48 h virtual follow‐up with the MOS nurse practitioner. Further instructions were offered by the nurse practitioner if there were any concerns of complications or dehydration, for instance, presentation to the emergency department or attending outpatient infusion services for intravenous hydration. Any postoperative problems at this stage were communicated directly to the treating surgeon and MOS medical lead.

**TABLE 2:** Criteria‐led discharge of day‐case bariatric surgery.

Part A: medical review	Yes	No
Review note from surgeon		

Part B: multidisciplinary discharge criteria	Yes	No
1. Tolerating fluids 2a.Controlled pain 2b. Discharge script pain relief provided 3a. Controlled nausea 3b. Discharge script antiemetic provided 4. Discharge script proton pump inhibitor provided 5. Discharge script anticoagulation as needed 6. TEDS in situ 7. Wound and dressing clean and dry 8. Incentive spirometry 9. Cleared by MOS		

Part C: patient criteria	Yes	No
All observations between the flags or within acceptable limits for this patient		
Has not required a rapid response for the patient in the last 24 h		
Nursing discharge checklist completed		
Medical certificate provided		

### 2.5 Outcomes and Statistical Analysis

Clinical data for selected patients were extracted from the database. Only patients who were intended for SDD were included. The primary outcome of this study was the number of patients that achieved successful SDD. Secondary outcomes comprised 30‐day readmission rates, complications (< 30 days), outpatient management, and mortality.

All statistical analyses were performed using SPSS for Windows version 24 (IBM Corporation, New York, USA). Patient characteristics were described using frequency and descriptive analyses. Ethics approval was granted by Greater Western Human Research Ethics Committee (ETH01585).

## 3 Results

There were 38 patients intended for the SDD pathway, with female predominance (*n* = 33/38; 86.8%). The majority was of non‐Aboriginal and Torres Straits Islander (*n* = 30/38; 78.9%) and EOSS stage 2 (*n* = 25/38; 65.8%). The median age and preoperative BMI were 39.5 (22–64) and 49.3 (37.6–69.4), respectively. LSG was the most common procedure performed (*n* = 26/38; 68.4%) followed by RYGB (*n* = 10/38; 26.3%) and OAGB (*n* = 2/38; 5.3%). Comorbidities are presented in Table 3. In this cohort, the median operative duration was 90.5 min (range 65–141 min) and the median length of stay was 0 days (range 0–1 day).

**TABLE 3:** Baseline characteristics of patients intended for SDD bariatric surgery.

Variables	Total patients (*n* = 38)
ATSI (%)	
Yes	8 (21.1)
No	30 (78.9)
Gender (%)	
Female	33 (86.8)
Male	5 (13.2)
Comorbidities (%)	
Diabetes	11 (28.9)
HTN	7 (18.4)
Chol	1 (2.6)
OSA	8 (21.1)
NASH	11 (28.9)
GORD	18 (47.4)
Type of surgery (%)	
LSG	26 (68.4)
RYGB	10 (26.3)
OAGB	2 (5.3)
Smoking (%)	
No	27 (71.1)
Yes	0
Previous smoker	11 (28.9)
EOSS (%)	
0	0
1	11 (28.9)
2	25 (65.8)
3	2 (5.2)
Median age (range)	39.5 (22–64)
Median LOS (range)	0 day (0–1)
Median BMI (range)	49.3 (37.6–69.4)

Abbreviations: ATSI, aboriginal torres straits islanders; BMI, body mass index; GORD, gastro‐oesophageal reflux disease; HTN, hypertension; LOS, length of stay; LSG, laparoscopic sleeve gastrectomy; NASH, non‐alcoholic steatohepatitis; OAGB, one‐anastomosis gastric bypass; OSA, obstructive sleep apnea; RYGB, Roux‐en‐Y gastric bypass.

Thirty‐five patients (92.1%) were successfully discharged on the day via the SDD pathway (Table 4). Three patients did not satisfy the criteria‐led discharge and were hospitalised overnight. Of the patients who were successfully discharged, one was readmitted within 30 days (2.9%) due to starvation ketosis. Meanwhile, four patients (11.4%) required intravenous fluid administration in the outpatient infusion clinic. Notably, there were neither recorded surgical complications nor any 30‐day reoperations, returns to theatre, or mortalities.

**TABLE 4:** Postoperative outcomes of patients intended for SDD bariatric surgery.

Outcomes	Total patients (*n* = 38)
Successful SDD (%)	35 (92.1)
Readmission of SDD bariatric patient (%)	1 (2.9) – Starvation ketosis requiring intravenous therapy (1 patient)
Outpatient treatment required for SDD bariatric patient	4 (11.4%) – Intravenous therapy (4 patients)
Median duration of surgery (range)	90.5 min (65–141)
Surgical complications	None
30‐day reoperation	0
30‐day return to theatre	0
30‐day mortality	0

Abbreviation: SDD, same‐day discharge.

## 4 Discussion

There is significant inequality in the uptake of MBS among the obese population, despite its well‐documented benefits in managing obesity and related complications [2, 3, 4]. This disparity is largely attributed to out‐of‐pocket costs, the lack of private health insurance [17] and the lack of publicly accessed obesity services with access to MBS [18]. Additionally, the COVID‐19 pandemic exacerbated the issue by causing delays in elective bariatric procedures due to resource reallocation [19]. In response, the Australian and New Zealand Metabolic and Obesity Surgery Society (ANZMOSS) developed a National Framework aimed at facilitating the implementation of MBS within the public health system [8]. Expanding access to MBS could deliver substantial health benefits to many Australians; however, securing sufficient funding remains a significant challenge [6]. A potential solution to cost containment lies in shifting postoperative care from overnight hospitalisation to SDD, which may encourage policymakers to improve the provision of publicly funded MBS. Our study—the first to evaluate SDD MBS in an Australian public, regional hospital—demonstrates its feasibility and safety in this resource‐constrained setting. At our institution, the SDD protocol (initiated in 2022) enabled approximately 30% of eligible public patients to undergo surgery with a 92.1% same‐day discharge success rate, a 2.9% readmission rate, and no 30‐day reoperations or mortality. Importantly, there were no instances of 30‐day reoperation, return to theatre, ICU admission, or mortality in our cohort.

The model of care for outpatient laparoscopic procedures was popularised with surgeries, such as cholecystectomy [20] and inguinal hernia repair [21]. SDD MBS builds upon the success of ambulatory surgical care to minimise postoperative hospital stay. This approach initially gained traction with laparoscopic adjustable gastric banding in Europe and the United States, where it was shown to be both safe and feasible as a same‐day procedure [22, 23]. Subsequently, interest grew in extending this model to other bariatric procedures, including LSG and RYGB. Billing et al. conducted 250 cases of LSG at an ambulatory surgical centre, reporting favourable outcomes. The success rate of SDD was 99.2% with a readmission rate of 3.6%. One case of staple line leak was reported, which was successfully repaired laparoscopically [14]. A separate meta‐analysis [24], which included 14 studies comprising 33 403 patients who underwent LSG and RYGB, reported a weighted pooled success rate for SDD of 99%. The overall morbidity was 4%, with a readmission rate of less than 4% and a weighted pooled leak rate of 1%. Results from our study are consistent with the current literature and underscore the potential for broader adoption of SDD in MBS locally.

A review of our patients revealed that two out of three failed the SDD pathway due to inadequate fluid intake caused by nausea. Similarly, in a large series of 500 SDD MBS patients, nausea was identified as one of the most common reasons for overnight hospitalisation following RYGB [9]. Another prospective study confirmed these findings, with nausea and vomiting being the primary reasons for failed SDD after LSG [15]. Postoperative nausea and vomiting (PONV) are significant barriers to safe discharge in this cohort, as they can restrict oral intake, resulting in dehydration, electrolyte imbalances, prolonged hospital stays, and readmissions [25]. These challenges exist due to surgical, patient, and anaesthetic factors. The American Society for Metabolic and Bariatric Surgery (ASMBS) addressed this issue by releasing a position statement advocating for evidence‐based pharmacologic and nontraditional antiemetic prevention and treatment strategies [25]. This highlights the importance of incorporating enhanced perioperative management protocols, such as prophylactic antiemetics and multimodal analgesia, into SDD pathways. In our practice, these strategies form a cornerstone of our protocol. A dedicated bariatric anaesthetist is involved in the surgery, employing an opioid‐sparing approach to minimise PONV. Additionally, we place a strong emphasis on preoperative counselling to educate patients about realistic surgical expectations and the importance of timely antiemetic and pain management interventions following discharge.

We encountered a case of readmission due to starvation ketosis following day‐only MBS. This was not entirely unexpected, as dehydration is a well‐documented factor associated with readmissions following bariatric procedures [26, 27]. Oral hydration has been shown to decrease significantly after surgeries such as LSG and RYGB [28], primarily due to the dramatic reduction in gastric volume and altered gut hormone secretion. These changes result in a more rapid sensation of gastric fullness, which can significantly impact fluid intake [29]. Additionally, inadequate postoperative dietary guidance may contribute to these issues. A national survey in Sweden revealed that patients often received conflicting advice regarding postoperative diets [30], with a common misconception being the need to avoid liquids during meals to control calorie intake. In contrast, research highlights the benefits of dietary counselling, showing fewer episodes of nausea, vomiting, and gastric dumping among patients who underwent RYGB with adequate dietary guidance [31]. At our institution, we address these challenges through regular dietetics consultations both before and after surgery. This approach helps dispel dietary confusion and promotes consistent adherence to postoperative dietary and fluid requirements, reducing variability in eating and drinking habits. We strongly advocate for the integration of professional dietary advice into pre‐ and post‐operative care to improve compliance with fluid intake and minimise the risk of readmission.

In our experience, four patients required intravenous fluid administration in the outpatient infusion clinic, all of whom were successfully managed in the community without further complications. This success was largely attributed to the efforts of our diligence of the MOS nurse practitioner, who plays a critical role in providing regular follow‐up care and ensuring timely coordination and intervention when necessary. Importantly, the nurse practitioner also served as a key point of contact for postoperative concerns, providing a‘safety net’ through coordination of review via the outpatient infusion service, which enabled prompt assessment while avoiding unnecessary emergency department presentations. This highlights the importance of a streamlined, multidisciplinary approach in preventing complications and ensuring optimal outcomes following MBS. This underlines the importance of a streamlined, multidisciplinary approach to prevent complications and ensure optimal outcomes following MBS. While the availability of a dedicated specialist nurse enhanced the feasibility and safety of our SDD model, we acknowledge that SDD may still be achievable in centres without such resources through structured protocols. These might include the use of remote monitoring devices for early detection, planned review by a surgical registrar, or clear patient instructions outlining when to seek emergency care. However, implementing such systems without dedicated staff may pose challenges, and we recognise that the provision of a reliable safety net is heavily dependent on available funding and staffing models.

The results of our study demonstrate an encouraging safety profile and the feasibility of SDD MBS, consistent with findings from contemporary literature across Europe and the United States [9, 12, 13, 15, 16, 24]. However, some authors have reported unfavourable outcomes with SDD [32, 33]. For instance, Inaba and colleagues [33] analysed a national database to compare outcomes between same‐day and first‐postoperative‐day discharge after LSG. They concluded that SDD was associated with higher overall morbidity, readmission, and reoperation rates. These findings contradict our results, potentially for two reasons. First, a significant proportion of patients in the SDD group (30%) had sleep apnoea and at least 1.3% had a cardiac history. Second, it was unclear whether these patients followed a specific pre‐ and post‐operative day‐case protocol.

Based on these observations, we propose that two critical elements are essential for the successful implementation of SDD: (1) rigorous patient selection with clear discharge criteria; and (2) the use of a dedicated SDD protocol.

Careful patient selection is paramount. The most important part of this is patient engagement. Before a patient is selected for SDD, they have a frank conversation about the risks and benefits of this so that there is shared decision making at all points. The patient is then evaluated from a medical perspective, looking for contraindications, including poorly controlled or undiagnosed sleep apnoea, unstable coronary artery disease and unstable diabetes. Good patient evaluation, engagement and support form the backbone of a successful SDD program. In our cohort thus far, approximately 25%–35% of the total MOS cohort are eligible. In the future, this percentage may increase as we fine tune our selection criteria. We have started this program with very conservative criteria to maximise safety.

The adoption of an enhanced recovery after surgery protocol is imperative for the success of SDD MBS. This approach relies on a multidisciplinary and multimodal framework to optimise patient management. Key components include patient education, opioid‐sparing multimodal analgesia, prophylaxis for postoperative nausea and vomiting, and goal‐directed fluid therapy [34]. At our institution, our MOS team includes surgeons, a bariatric anaesthetist, a metabolic physician, a nurse practitioner, dietitians, and psychologists. Standardised care is provided from the preoperative to postoperative period to optimise recovery and minimise perioperative surgical stress. Patient education plays a central role in successful SDD, ensuring patients understand fluid intake requirements, postoperative mobilisation, and potential complications. Well‐informed patients are more motivated to adhere to instructions and seek medical help promptly if needed. Scheduled early follow‐up enables expeditious interventions, such as outpatient intravenous fluid therapy, to address complications.

This is the first published study in Australia to evaluate the safety and feasibility of SDD in MBS. These outcomes underscore SDD's potential to address systemic inequities in rural and public healthcare systems, where bed shortages and funding limitations disproportionately restrict access. By optimising resource utilisation, this model provides a replicable framework for other Australian hospitals to scale services within existing public funding structures. However, our study has limitations. It is a retrospective cohort study, and selection bias may be introduced inadvertently. Additionally, there is an inherent selection bias as lower‐risk patients are more likely to be offered and planned for SDD. The sample size is also small due to the limited number of publicly funded MBS performed annually in our program. As a result, the findings should be interpreted cautiously. Resource limitations, such as the absence of remote monitoring devices, constrained our ability to monitor patients' vital signs in real time. Implementing remote monitoring could provide prompt medical advice for patients experiencing deviations in their vital signs. Finally, our study did not assess the financial implications of SDD MBS. Future research should include an economic evaluation to better understand the cost‐effectiveness of this approach.

## 5 Conclusion

In conclusion, SDD MBS appears to be a safe and feasible approach, demonstrating low rates of readmission and complications. The success of such a program depends on careful patient selection, strict discharge criteria, and the implementation of a dedicated SDD protocol. This model has the potential to increase the accessibility of publicly funded MBS services nationwide. However, further multi‐centred studies are needed to assess the financial implications of SDD on the healthcare system and to draw more definitive conclusions.

## Conflicts of Interest

The authors declare no conflicts of interest.

## Data Availability

Research data are not shared.
